# First-in-human administration of [^61^Cu]Cu-NODAGA-LM3 and head-to-head comparison with [^68^Ga]Ga-DOTA-TOC PET/CT as part of the phase I/II COPPER PET in NET study

**DOI:** 10.1007/s00259-025-07301-3

**Published:** 2025-05-24

**Authors:** Guillaume P. Nicolas, Manuel Alejandre Lafont, Lisa McDougall, Felix Kaul, Alin Chirindel, Wolfgang Weber, Angela Llmazares, Anass Johayem, Markus Baier, Nicole Schubert, Francesco De Rose, Leila Jaafar-Thiel, Andreas Bauman, Melpomeni Fani, Damian Wild

**Affiliations:** 1 Division of Nuclear Medicine, University Hospital Basel, Basel, Switzerland; 2 Division of Radiopharmaceutical Chemistry, University Hospital Basel, Basel, Switzerland; 3 ENETS Center of Excellence for Neuroendocrine and Endocrine Tumours, University Hospital Basel, Basel, Switzerland; 4 Department of Nuclear Medicine at “Klinikum rechts der Isar”, Technical University of Munich, Munich, Germany; 5 Centre for Radiopharmacy, Department of Nuclear Medicine, University Hospital of Zurich, Zurich, Switzerland; 6 Nuclidium AG, Basel, Switzerland

We present the first patient in the prospective, single-centre, randomized, cross-over phase I/II study (COPPER PET in NET – NCT06455358) investigating [⁶¹Cu]Cu-NODAGA-LM3 for PET/CT or PET/MRI imaging.

While [⁶⁸Ga]Ga-labelled somatostatin receptor (sstr) agonists perform well clinically, they face limitations: competing access to ^68^Ga generator, short half-life, and suboptimal spatial resolution. High physiological uptake in the liver, gastrointestinal tract, and pancreas complicates neuroendocrine tumour (NET) detection. Sstr antagonists like [⁶⁸Ga]Ga-NODAGA-JR11 offer superior lesion detection (93.7% vs. 59.2% sensitivity) [1], yet ⁶⁸Ga’s constraints remain. Cyclotron-produced [⁶¹Cu]Cu-labelled sstr antagonists overcome these challenges, enabling higher production capacity, broader accessibility, and improved imaging performance [2].

A 70-year-old man with a grade 2 small intestinal NET (Ki-67 4%) and pulmonary, hepatic, and nodal metastases underwent [⁶¹Cu]Cu-NODAGA-LM3 and [⁶⁸Ga]Ga-DOTA-TOC PET/CT seven days apart, post-lanreotide (28 and 21 days, respectively). Imaging was performed on the same scanner with identical reconstruction protocols.

The figure compares PET/CT images of [⁶¹Cu]Cu-NODAGA-LM3 (140 MBq, specific activity ~ 12 MBq/nmol) and [⁶⁸Ga]Ga-DOTA-TOC (103 MBq, ~ 5 MBq/nmol). [⁶¹Cu]Cu-NODAGA-LM3 showed higher tumour uptake (SUVmax 25.4 vs. 23.2; mean SUVmean of 12.2 vs. 9.1 of the 4 matching liver lesions (horizontal white arrows)) and lower liver (SUVmax 3.6 vs. 6.5), spleen (9.3 vs. 22.3), and intestine (3.8 vs. 4.5) background, improving tumour-to-background contrast (7.1 vs. 4.2). Additional small liver (vertical white arrows) and pulmonary (red arrow) metastases were detected. Discrepant findings will be resolved using biopsy and/or composite imaging during 2–7 months of follow-up as the gold standard. Sensitivity will be tested for non-inferiority using a mixed-effects logistic regression model. Safety will be assessed using the Common Terminology Criteria for Adverse Events version 5.0.

**Figure:**
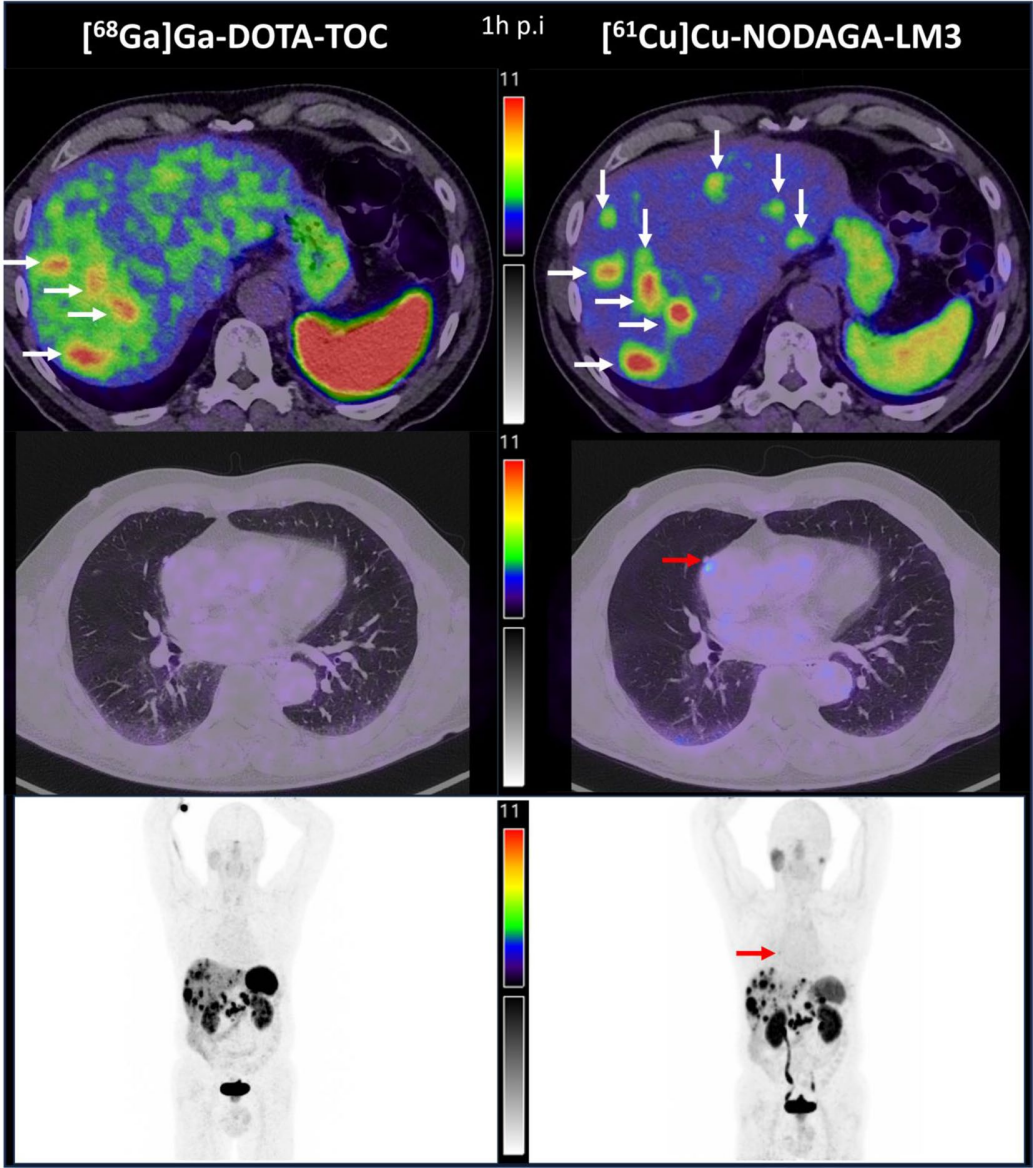

## Data Availability

Raw data will be made available upon reasonable request.

## Declarations

### Clinical trial registration

This study is registered with ClinicalTrials.gov (NCT06455358).
